# Increasing frequency of combination medical therapy in the treatment of acromegaly with the GH receptor antagonist pegvisomant

**DOI:** 10.1530/EJE-17-0996

**Published:** 2018-01-25

**Authors:** Christian J Strasburger, Anders Mattsson, Patrick Wilton, Ferah Aydin, Judith Hey-Hadavi, Beverly M K Biller

**Affiliations:** 1 Department of Medicine for EndocrinologyDiabetes and Nutritional Medicine, Charité Universitätsmedizin, Campus Mitte, Berlin, Germany; 2 Endocrine CarePfizer Health AB, Sollentuna, Sweden; 3 Endocrine CarePfizer Health AB, Stockholm, Sweden; 4 Endocrine CarePfizer Inc., New York City, New York, USA; 5 Neuroendocrine UnitMassachusetts General Hospital, Boston, Massachusetts, USA

## Abstract

Pegvisomant monotherapy is effective and safe in treatment of acromegaly. However, some clinicians combine pegvisomant with somatostatin analogues (SSA) or dopamine agonist (DA). In this analysis of ACROSTUDY, a long-term non-interventional study, the use of combination regimens was evaluated. Based on their baseline treatment, 2043 patients were retrospectively categorized as: long-acting SSA combined with pegvisomant, ‘Combo SSA’ 768 patients (38%); DA combined with pegvisomant, ‘Combo DA’ 123 (6%); pegvisomant monotherapy, ‘Peg mono’ 1128 (55%). Treatment patterns changed over the 10-year period, with recent patients more likely to receive any combination (20% in 2003 vs 54% in 2012). Combo SSA use varied widely among countries from 22% to 78%. Exposure periods of the three treatment modalities were defined from pegvisomant start until the last visit in ACROSTUDY; patients could switch treatment categories. At year 4, IGF-I was normal in 62% of Combo SSA, 63% of Combo DA and 65% of Peg mono groups. Pegvisomant was initiated as daily injections in 94% of patients in the Peg mono group, 66% of Combo SSA and 91% of Combo DA patients. During 6169 years of treatment exposure, 3424 adverse events (AEs) were reported in 946 (51%) patients, of which 617 (18%) were serious and 401 (12%) were considered treatment related. The reported incidence of serious AEs and treatment-related non-serious AEs were similar among the three treatment modalities. This analysis describes real-world clinical care and shows favorable efficacy and safety for Peg mono and combinations. Novel findings include an increased use of combination therapy over time and variability in treatment modalities between countries.

## Introduction

While pituitary surgery remains the first-line treatment for acromegaly, medical therapy is needed for about 50% of patients who are not cured by surgery alone (1, 2, 3). Medical treatment options for residual disease or for those patients ineligible for surgery include somatostatin analogues (SSAs), dopamine agonists (DA) and the growth hormone (GH) receptor antagonist pegvisomant (1, 2). Pegvisomant monotherapy has been shown to normalize IGF-I levels in 63–97% of patients (4, 5, 6, 7). The efficacy of SSA monotherapy varies, and it has been reported that 17–55% of acromegaly patient had normal IGF-I levels on this treatment (8, 9, 10, 11). Patients who do not demonstrate a satisfactory response to SSA treatment may be switched to pegvisomant monotherapy (4). Some clinicians, however, may decide to treat patients who fail to achieve adequate control on SSA therapy with a combination of SSA and pegvisomant rather than substituting pegvisomant for the SSA, to combine the beneficial effects of both drugs (4, 13, 14). There are limited controlled clinical trial data to guide dosing and titration when pegvisomant is combined with SSA treatment (14, 15, 16, 17, 18, 19).

In ACROSTUDY, a long-term global non-interventional study, initiated in 2004 documenting clinical practice in acromegaly patients treated with pegvisomant, it was noted that a substantial number of patients were treated with combination therapy (4, 5). The present analysis investigates those patients in ACROSTUDY who were treated with pegvisomant along with at least one other medication for acromegaly, in order to learn more about combined medical treatment in real-world practice.

## Subjects and methods

Descriptions of ACROSTUDY study methods have been published in more details previously (4, 5, 20).

All patients enrolled in ACROSTUDY by November 17, 2014 were retrospectively classified in three main categories based on their medical therapy for acromegaly at ‘Baseline’, defined as the date of initiation of pegvisomant. In 76% of the patients, pegvisomant had been initiated before enrolment in ACROSTUDY. The three main medical treatment modalities were: (1) long-acting somatostatin analog in combination with pegvisomant, named, ‘Combo SSA’; (2) dopamine agonist in combination with pegvisomant, named ‘Combo DA’; (3) pegvisomant monotherapy, referred to as ‘Peg mono’. Patients receiving both SSA and DA in addition to pegvisomant were included into the Combo SSA group. A few patients with other combinations of medications (for example, use of pegvisomant with short-acting subcutaneous octreotide) were excluded from the present analysis due to few observations. Also excluded from analyses were periods/time points when patients had switched to a therapy with SSA only or DA only after pegvisomant start. Patients could be switched between treatment modalities at any time by the prescribing physicians. To address this complexity, the analysis defined ‘exposure periods’ of the 3 different treatment modalities to account for the fact that the same patient may have been exposed to 1 or 2 or 3 of these treatments after starting on pegvisomant. Therefore, an individual patient could be in different treatment ‘exposure period’ categories at different times during follow-up. Those patients who changed exposure during follow-up were subsequently classified by the type of treatment being administered at the date of the yearly visits, each of which represented a cross-sectional analysis point. The yearly visit was defined as the clinical visit closest to each 12-month time point after the baseline date, with a plus/minus six-month time interval around that date. Because long-acting SSA injections are not always administered at exactly 4-week intervals, patients were defined as being in the Combo SSA category as long as the latest SSA injection was within the six weeks preceding a visit date. Patients in the Combo DA category must have received a dose of DA within 30 days before the date of classification. To be included in the Peg mono category, no SSA or DA was allowed within 42 or 30 days, respectively. Pegvisomant dosing schedules that were less frequent than daily were transformed to daily dose equivalents before evaluation. Treatment and treatment effects, including the dose of pegvisomant and percentage of patients with IGF-I levels within normal limits, are presented as yearly cross-sectional values based on all available patients still in the same treatment category from pegvisomant start until a treatment switch occurred, or if no switch occurred, until the last reported visit.

Reasons for use of combination therapy of SSA or DA with pegvisomant were recorded by the investigator for 416 patients on an optional report form. Choices were listed and included: IGF-I not controlled, headache, hyperprolactinemia, tumor near optic chiasm, prolactin co-expression, cost or other. The options were answered by investigators as ‘yes’ or ‘no’; more than one response was allowed.

Serum IGF-I levels and liver tests were measured at local laboratories as previously reported (4, 5, 20). Pituitary MRI scans were to be sent for central assessment if the local radiologist reported a significant change in pituitary volume irrespective of the clinical significance of the change (5).

Safety was further analyzed by evaluating all adverse events that were reported after enrolment in ACROSTUDY (4, 5, 20). Adverse events (AEs) were attributed to the medical treatment modality exposure period during which they occurred (Combo SSA, Combo DA, Peg mono). All AE reports from the investigators were coded by the data manager according to Medical Dictionary for Regulatory Activities (MedDRA 14.1).

### Statistical methods

The treatment exposure periods for each patient were mapped out in detail, from pegvisomant start until the last known treatment date in ACROSTUDY. The different exposure periods were classified based on the three main treatment modalities: (1) Combo SSA, (2) Combo DA, (3) Peg mono. Additionally, (4) no dose (i.e. no exposure to pegvisomant, SSA, or DA) and (5) other (which could be exposure to SSA only, DA only, SSA + DA only, pegvisomant + SSA short-acting, or other) were identified. Through this approach, treatment-exposure status at any given date between pegvisomant start (baseline) and the last reported date in ACROSTUDY was precisely determined and assigned. Only data during periods when patients were exposed to pegvisomant in the categories Combo SSA or Combo DA or Peg mono were included in the analyses. Some patients switched between different treatment exposures. For example, a patient could switch from Peg mono to Combo SSA, or to Combo DA or vice versa at any time point. For analyses of effectiveness (based on serum IGF-I) and safety (liver tests and pituitary imaging), treatment exposure status was determined at yearly visits as defined earlier. Patients who switched treatment may have contributed to AEs in more than one treatment exposure category, which makes the categories non-independent. Due to these switches, an event may be related to circumstances that occurred during an earlier exposure period, when a patient may have been on a different treatment combination. No incidence rate ratios or tests were thus performed.

Total numbers of treatment-exposure years were calculated as the sum of years on the treatment combination when the dose was >0 for the respective drug (SSA, DA, pegvisomant) from ACROSTUDY start to last reported date.

### Study population

**Figure 1:**
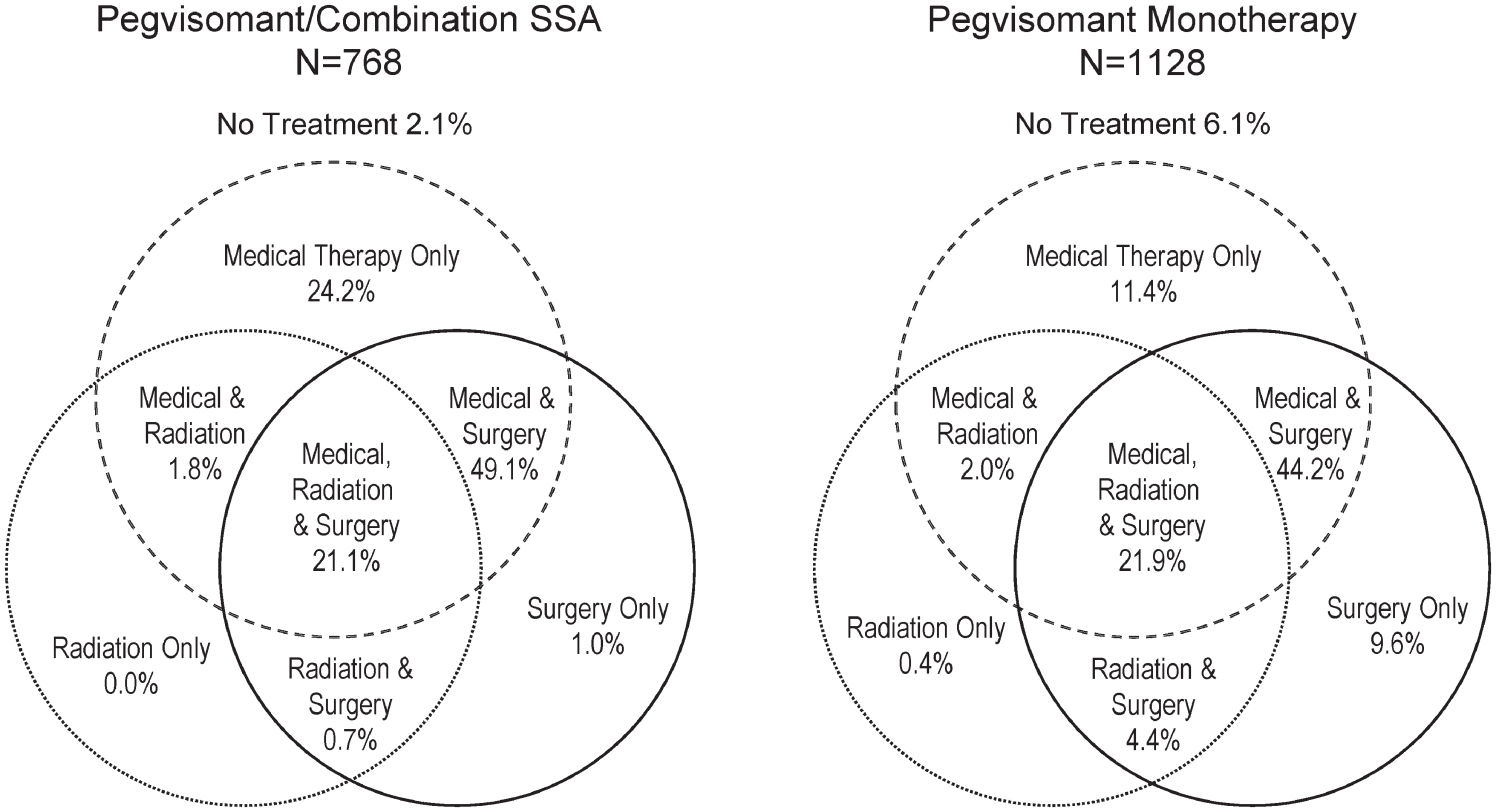
Treatment for acromegaly before pegvisomant start.

As of the data cut in November 17, 2014, a total of 2043 patients were enrolled in ACROSTUDY. The majority (77%) had undergone pituitary surgery and in one-third of these, radiotherapy was also administered. Figure 1 represents the different treatments prior to pegvisomant start in the Combo SSA and Peg mono categories. Background characteristics and demographics for each treatment category at baseline are presented in Table 1. The gender distribution, mean age, weight and BMI at baseline were similar between the three categories. Initiation of pegvisomant therapy was earlier than ACROSTUDY start for 75% of the patients, because the medication was available before the database opened for some participating countries.

**Table 1:** Background statistics by treatment category* at pegvisomant start in 2019 patients enrolled in ACROSTUDY**. Data are presented as *n* (%) or as median ((5th–95th percentile)

	Combo SSA	Combo DA	Peg mono	All
Total number	768 (38.0)	123 (6.1)	1128 (55.9)	2019 (100.0)
Male	411 (53.5)	59 (48.0)	564 (50.0)	1034 (51.2)
Female	357 (46.5)	64 (52.0)	564 (50.0)	985 (48.8)
Tumor treatment				
Surgery, no RT	385 (50.1)	51 (41.5)	606 (53.7)	1042 (51.6)
Surgery + RT	167 (21.7)	48 (39.0)	297 (26.3)	512 (25.4)
RT, no surgery	14 (1.8)	4 (3.2)	27 (2.4)	45 (2.2)
No Surgery, no RT reported	202 (26.3)	20 (16.3)	198 (17.6)	420 (20.8)
Previous medical treatment				
SSA, no DA	521 (67.8)	7 (5.7)	581 (51.5)	1109 (54.9)
SSA + DA	208 (27.1)	86 (69.9)	277 (24.6)	571 (28.3)
DA, no SSA	10 (1.3)	30 (24.4)	38 (3.4)	78 (3.9)
No SSA, no DA	29 (3.8)	0 (0.0)	232 (20.6)	261 (12.9)
Diabetes mellitus				
All	250 (32.6)	43 (35.0)	339 (30.0)	632 (31.3)
Hypertension				
All	404 (52.6)	74 (60.2)	619 (54.9)	1097 (54.3)
Sleep apnea				
All	123 (16.0)	20 (16.3)	215 (19.1)	358 (17.7)
Age (years)				
All	49 (26–72)	51 (27–73)	50 (27–73)	50 (26–73)
Weight (kg)				
Male	94 (70–130)	97 (75–126)	96 (74–132)	95 (73–130)
Female	75 (58–109)	80 (57–115)	75 (57–112)	75 (57–110)
All	85 (60–121)	90 (60–122)	87 (61–125)	87 (61–123)
BMI (kg/m^2^)				
All	28 (22–38)	30 (22–42)	29 (22–40)	29 (22–40)
Years since diagnosis				
All	3.4 (0.6–23)	7.4 (1.1–28)	4.3 (0.5–26)	4.2 (0.5–25)

*Peg mono, pegvisomant mono; Combo SSA, pegvisomant + somatostatin analogues ± dopamine agonists; Combo DA = pegvisomant + dopamine agonists; **24 patients were classified as other and were mainly treated with pegvisomant plus SSA SA, and therefore excluded from this presentation.

## Results

### Patient characteristics

Based on the retrospective classification of medical treatment at pegvisomant start, there were 768 patients (38%) on Combo SSA, 123 (6%) on Combo DA, 1128 (55%) on Peg mono and 24 (1%) on other treatment. In the Peg mono category, 20.6% had not been reported with any medical therapy for acromegaly before pegvisomant start, while this was 3.8% for the Combo SSA category (Table 1). The time since diagnosis of acromegaly at the initiation of pegvisomant treatment appears to be shorter in the Combo SSA category than in the Peg mono and Combo DA categories (Table 1). However, the majority of these patients were treated with surgery and/or radiotherapy. Treatment exposure time was 2179 years in the Combo SSA category, and 412 and 3578 years in the Combo DA and Peg mono categories respectively.

In the 571 patients with seven years of follow-up, 55% remained in the original treatment category, whereas the rest had switched therapy at least once, which could have happened at any time point. The percentages of patients remaining in their original treatment category at seven years after pegvisomant start were: Combo SSA 44%, Combo DA 38% and Peg mono 64% (Supplementary Table 1, see section on supplementary data given at the end of this article). Overall, diabetes mellitus as comorbidity was found in 31% (range: 30 to 35%) of patients at pegvisomant start, while 54% (range: 52–60%) of patients reported hypertension (Table 1).

### Reason for switch to combination therapy

**Figure 2:**
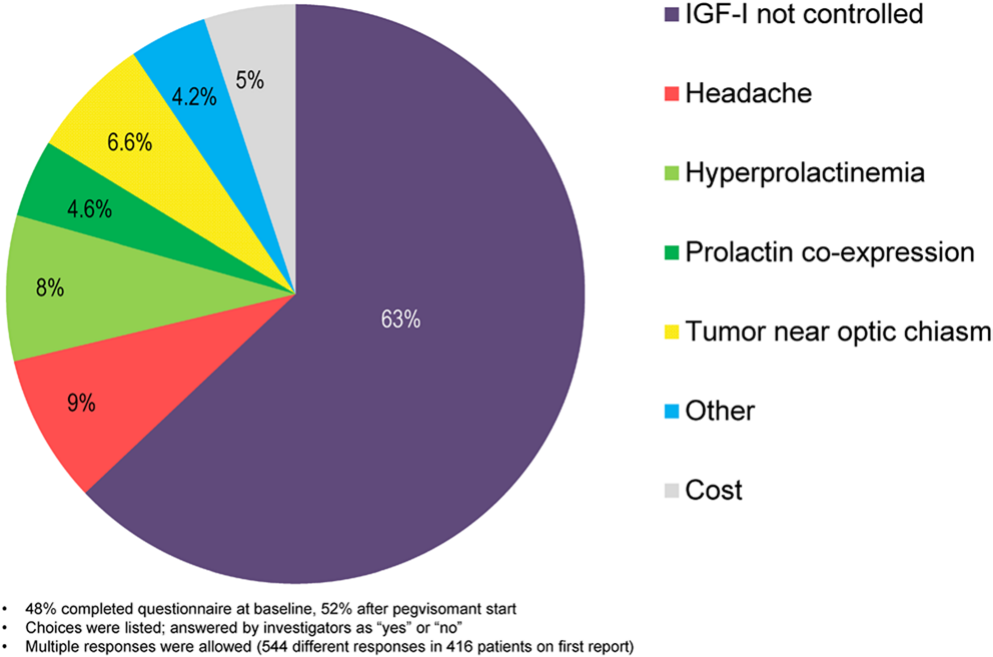
Responses to ‘reason for use of combination therapy’ recorded for 416 patients treated with Combo SSA or Combo DA. Data from earliest (first) case form reported. 48% completed questionnaire at baseline, 52% after pegvisomant start. Choices were listed; answered by investigators as ‘yes’ or ‘no’. Multiple responses were allowed (544 different responses in 416 patients on first report).

After 7 years of follow-up, according to the exposure period mapping, 45% of patients had switched treatment categories at least once. Data regarding the reason(s) for combining pegvisomant with another medication were available for 416 patients (349 on Combo SSA and 52 on Combo DA, 15 missing information). Lack of efficacy (IGF-I not controlled) was the most common reason given for using more than one medication. Other reasons included headache, hyperprolactinemia/prolactin co-expression, tumor near optic chiasm and cost (Fig. 2).

### Treatment modalities at pegvisomant start by calendar year and country

**Figure 3:**
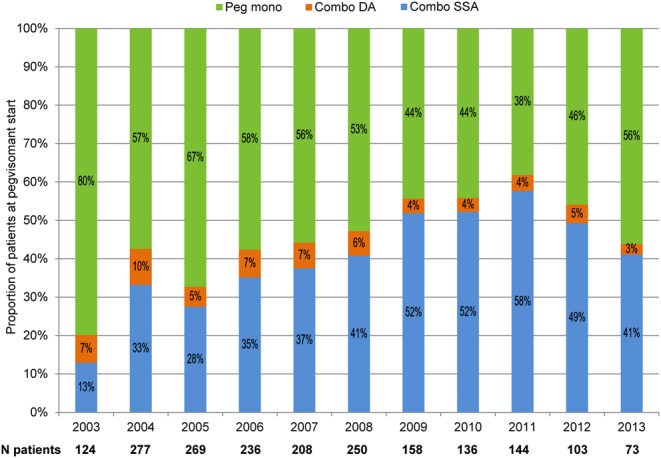
Treatment modalities at pegvisomant start by calendar year.

**Figure 4:**
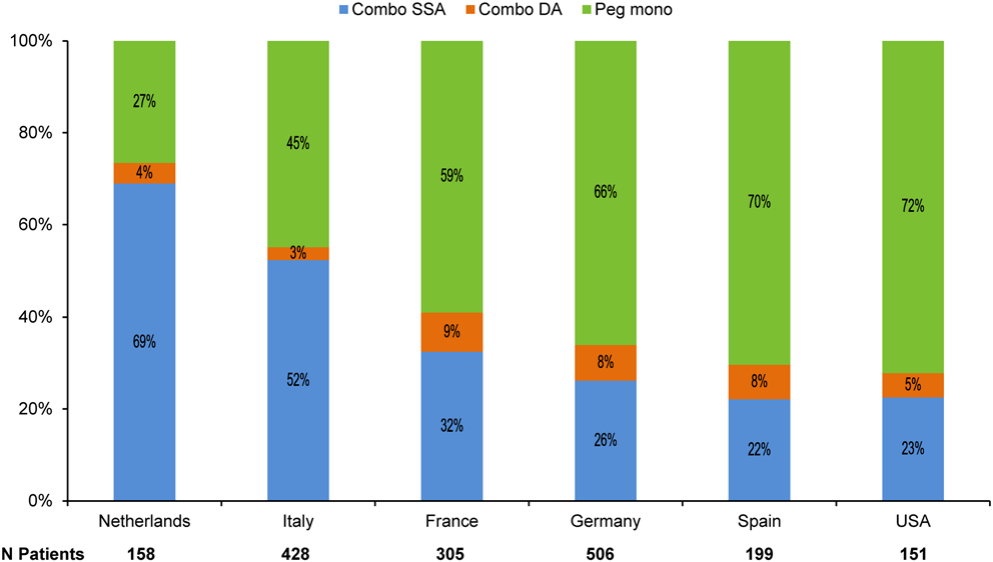
Treatment modalities at pegvisomant start in countries with over 100 ACROSTUDY patients.

Overall treatment patterns in ACROSTUDY changed as the study progressed, with a higher percentage of patients receiving combination therapy over time. In 2003, 20% of patients received Combo SSA or Combo DA at the start of ACROSTUDY enrolment compared to 54% in 2012 (Fig. 3). There were also notable differences in prescribing practices between countries. Pegvisomant in combination with long-acting SSA was commonly used in the Netherlands and Italy (69 and 52% of patients, respectively), whereas Peg mono was common in the USA, Spain and Germany (72, 70 and 66% of patients, respectively) (Fig. 4).

### Effectiveness

The mean daily doses, according to whether the IGF-I/ULN was normal or elevated at follow-up visits are shown in Supplementary Table 2. In general, within all groups, the mean pegvisomant dose increased slightly over time, but not all patients were titrated sufficiently. The percentages of patients with IGF-I within normal limits are presented for those who remained in the same original treatment modality after pegvisomant start in Supplementary Table 3. For example, at year 4, when the total number of patients remaining in their original treatment category was 492, this included 62% with normal IGF-I in the Combo SSA, 63% in the Combo DA and 65% in the Peg mono category (Supplementary Table 3). Overall treatment modalities, at baseline, pegvisomant was administered as a daily injection in 83% of the patients. In the Peg mono group, daily injections were used in 94%, while daily pegvisomant was used in 66% of Combo SSA patients and in 91% of Combo DA patients.

### Safety

#### Adverse events

For the safety analysis, a total of 1865 patients were evaluable for adverse events (AEs). During an overall 6169 years of exposure to the different treatments after ACROSTUDY start, 3424 AEs were reported in 946 (51%) patients, of which 617 (18%) were classified as serious and 401 (12%) were considered by the investigators as related to the treatment. There were 87 (4.7%) patients with drug withdrawn (temporarily, permanently, or dose delayed) due to serious AEs, regardless of whether they were considered by the investigator to be related to treatment. There were 48 deaths reported during exposure periods and none was considered to be treatment related by the investigators. The most commonly (>1.5% of patients) reported AEs (regardless of whether they were considered by the investigator to be related to treatment) were elevated IGF-I in 10.1% of the patients, followed by elevated liver transaminases (4.8%), headache (4.2%), vitamin D deficiency (3.6%), arthralgia (3.6%), osteoarthritis (2.7%), injection site reaction (3.0%), asthenia (2.1%), depression (1.9%), colonic polyps (1.8%), cholelithiasis (1.8%) and pituitary tumor recurrence (1.6%).

#### Liver tests

At pegvisomant start, ALT and AST were normal in 1026 of 1032 (99.4%) patients and in 1011 of 1013 (99.8%) patients with available results respectively, and abnormal (>3× ULN) in 6 (0.6%) and 2 (0.2%) patients, respectively. At least one follow-up liver test was reported after pegvisomant start in 1823 patients (90%). At the one year visit, two patients out of 894 (0.2%) with available samples had an ALT value >3× ULN. Hepatobiliary disorders and liver-related AEs were reported by the investigators using a variety of different terms in 162 patients; these included 32 with increased transaminases, 15 with increase of liver enzymes, 22 with abnormalities of ALT and 6 of AST. Other liver-related events included cholelithiasis (*n*=33), hepatic steatosis (*n*=12) and cholecystitis (*n*=6). As patients switched treatment modalities in between visits and laboratory data did not always coincide with a switch, it was not possible to separately evaluate liver test abnormalities over time related to treatment group.

#### Pituitary tumor assessment

Local MRI assessment of pituitary tumor size was reported at least once in 1816 subjects overall while 1647 had one or more image reported at least 30 days after the start of pegvisomant. When MRI investigations were available, tumor size decreases were reported by local radiologists in 323 patients (19.6%), increases in 119 patients (7.2%), while 54 (3.3%) showed both increase and decrease. Although all cases in which the local MRI assessment showed a change (whether read as smaller or larger) were to be submitted for central MRI readings, this was performed in just 228 cases. The additional central assessment reported a decrease in pituitary tumor size in 75 patients, an increase in 42, increase/decrease in 10, no change in 71, while in 30 there were insufficient data to make a determination (Supplementary Table 4). The local MRI readings that were submitted as showing an increase in tumor size (*n* = 50) were confirmed 20 cases as showing an increase, but were reclassified in 20 as showing no change and in 3 as having both an increase and decrease over time. Among these patients with locally reported decreases in tumor size (*n* = 80), central reading reported a decrease in 48, reclassified 24 as no change and 7 as showing an increase. Details of local vs central MRI reading classifications are summarized in Supplementary Table 4. Due to switching of treatment modalities at different intervals in between visits, it was not possible to separately evaluate MRI findings over time related to treatment group.

#### Injection site reactions

Administration site issues were reported in 51 (3%) of patients. The most common injection site reactions were reported as lipohypertrophy (*n* = 18), injection site reaction (*n* = 12) and lipodystrophy (*n* = 7). The number of reported injection site reactions was low across all treatment groups.

#### Glucose-related events

Adverse events of glucose homeostasis (regardless of whether they were considered by the investigator to be related to treatment) were reported in a total of 85 patients, regardless of whether or not they already had the diagnosis of diabetes. Diabetes mellitus/type 2 diabetes was reported as an AE in 50 patients, hyperglycemia/impaired glucose tolerance/impaired fasting glucose was listed in 15 patients and in 18 patients, increased glycosylated hemoglobin was noted.

## Discussion

The present analysis evaluated the long-term safety and efficacy of three different medical treatment approaches in patients with acromegaly being followed in ACROSTUDY. Patients who were prescribed a combination of pegvisomant and somatostatin analog therapy were evaluated as well as patients treated over the same years with pegvisomant monotherapy or with pegvisomant combined with a dopamine agonist. The current report uses a more recent data cut off, providing a larger cohort and including two additional years of enrolment and follow-up than the most recent ACROSTUDY publication, which was focused solely on pegvisomant monotherapy (4).

Two important and novel findings of this investigation were that the use of combination therapy at pegvisomant start became more common over the decade when new patients were being enrolled and that combination treatment varied considerably by geographic region. In the present report, the proportion of patients on any combination therapy enrolled in ACROSTUDY increased strikingly over time, from 20% of those enrolled in 2003 to 54% of patients enrolled a decade later in 2012.

Over a decade ago, the first clinical trial with a combination of pegvisomant and SSA conducted in a single center in the Netherlands was published (12), followed by longer studies showing IGF-I normalization rates of around 90% of patients (13, 14, 15). Other publications about combination therapy include limited numbers of patients with IGF-I normalization rates between 58 and 97% depending on how treatment and efficacy were defined (15, 16, 17, 18, 19). Neggers and coworkers reported that IGF-I was normal in 97% of 112 patients treated for a median of 4.9 years with SSA and once- or twice-weekly pegvisomant injections of 80 mg total per week (interquartile range 60–120 mg) (14). That was higher than in the present observational study, showing 61% of patients normalized IGF-I at five years with SSA and a mean pegvisomant daily dose of 17.9 mg. It is important to note that there are many differences between ACROSTUDY and the study reported by Neggers and coworkers (14). For example, a normal IGF-I was defined as below 1.2× ULN in that group of patients, in contrast to the stricter criterion of 1.0× ULN employed in the present study. In addition, the lowest IGF-I level achieved at any time during treatment was used to determine efficacy in the Neggers study, in contrast to the current report, where measurements taken at each one year interval were used. Other differences between the studies were that this report included 304 sites, whereas the Erasmus study reported on the experience at a single center and that most patients (66%) took pegvisomant daily in ACROSTUDY vs the once or twice-weekly dose administration schedule in the Neggers report. Finally, IGF-I levels were measured in local labs with a variety of assays during the present study, in contrast to the use of the Immulite 2000 in all patients in the Neggers study (14).

The combination of pegvisomant and dopamine agonists has been previously suggested to allow for a cost-effective combination therapy in acromegaly treatment. Higham and coworkers (21) reported achieving controlled IGF-I levels on 10 mg daily of pegvisomant in 68% of 24 patients treated in combination with a daily dose of 0.5 mg cabergoline, while after withdrawal of cabergoline only 26% maintained IGF-I within normal limits. To which extent this observation can be extrapolated to other acromegaly patient cohorts with potentially higher disease activity remains to be established. While dopamine agonists are indicated in cases of prolactin co-secretion by true somatomammotroph tumors or mixed adenomas in order to control the prolactin excess, its merit as co-medication for lowering the GH output by the adenoma appears mainly to be in patients with IGF-I levels not exceeding twice the upper limit of normal (22, 23).

The combination of cabergoline with pegvisomant is also discussed in a recent review by Kuhn and Chanson (24).

While it would be interesting to assess whether there was any ‘switch effect’ that might have impacted efficacy or safety, for many reasons (timing of IGF-I measurements, multiple switches in various directions over time, a global time trend to give higher pegvisomant doses), it would be a delicate task to address such a switch effect in a reliable statistical manner based on the data of this non-interventional observational study.

The effects of pegvisomant monotherapy in patients enrolled in ACROSTUDY were recently reported; after five years of treatment, 67.5% of patients had a normal IGF-I (4). The current report has a later data cut off with larger cohort and includes two additional years of enrolment and follow-up, with a normal IGF-I in the Peg mono group achieved in 65% of subjects with four years of treatment. The IGF-I normalization rates were similar in Combo SSA and Combo DA.

Pegvisomant is most often prescribed as daily subcutaneous injections. Over 90% of the Peg mono and Combo DA patients received daily injections, whereas in the Combo SSA group, 66% of patients received daily pegvisomant. Higham and coworkers (25) reported a small study in which some patients were successfully switched from daily to biweekly or weekly pegvisomant dosing. Other studies (14, 18) showed use of SSA in combination with less than daily pegvisomant dosing, which may have contributed to this practice in the real-world setting.

In previous publications, increased liver enzymes were noted to be more frequent in patients taking combination therapy compared to pegvisomant monotherapy (15, 16, 17). For the present analysis, it is difficult to determine whether or not combination treatment is associated with a higher rate of liver enzyme elevation because 45% of patients switched treatment groups at least once. However, the overall frequency of increased liver enzyme reports is lower than that in prior publications (6, 7). There are several possible reasons for this. It is possible that elevated liver tests did occur in some patients upon initiation of pegvisomant but prior to enrolment in ACROSTUDY and that patients who reached ACROSTUDY were considered by the investigators to be tolerating the medication. Additionally, as liver tests were only recorded yearly in many centers, transient elevations may not have been observed.

While treatment of somatotroph adenomas with SSAs is known to reduce tumor volume (1), the present analysis did not allow the evaluation of any difference between the combination of pegvisomant with SSA or DA and pegvisomant monotherapy due to switching of treatment modalities. After seven years, 45% of patients had switched at least once between treatments, so the ‘groups’ were not constant over time. It could also be a bias that patients with more difficultly in controlling disease were more likely to be switched from one modality to another. In the assessment of pituitary tumor volume 80% of patients had at least two MRI evaluations reported. By offering investigators the option to send MRI locally showing a change in pituitary volume, it was confirmed that tumor increases with pegvisomant treatment are uncommon as in previous reports (5).

In a small study, Jorgensen and coworkers (19) assessed the effects of cotreatment with pegvisomant and SSA on GH secretion, IGF-I levels and glucose tolerance. They reported that pegvisomant improved glucose tolerance in patients who failed to respond to SSA treatment. In ACROSTUDY, more than 30% of patients reported diabetes as a comorbidity at baseline, but in this type of observational study, changes in glucose homeostasis are hard to evaluate due to the infrequent reporting intervals and unclear causalities in those patients switching treatment modalities.

There are a number of limitations to this study design. Due to the observational nature of ACROSTUDY, patients were not randomized into treatment groups and the choice of which modality to use could have been affected by many things including investigator-, site- or country-specific typical practice. In addition, clinical factors in individual patients, such as tumor size and location, degree of biochemical disease activity and symptom severity may have produced uneven treatment cohort assignments. For example, it is possible that patients with more severe illness and/or larger tumors may have had a higher likelihood to be treated with combination therapy. In addition, 75% of patients were already being treated with pegvisomant before enrolment into ACROSTUDY, limiting the assessment of underlying disease severity. This also means that AEs experienced by patients upon initiation of pegvisomant would not have been captured if they occurred before ACROSTUDY enrolment. Additionally, the usual limitations of observational trials apply; while such studies are valuable because they reflect ‘real-life’ clinical care, data from patient visits are not gathered at pre-determined, regular time points as done in a prospective trial. As a result, data are sometimes missing at specified time points and adverse events may be underreported compared to clinical trials. A limitation regarding efficacy is that the doses of pegvisomant used in ACROSTUDY were often low; if higher doses had been administered, the rates of normal IGF-I might have been higher. It is also important to note that treatment categories were not independent; the same patient may have contributed information in more than one treatment category if management changed over time between monotherapy and combination therapy.

In conclusion, this is a large data set providing a description of the clinical care of patient treated with pegvisomant combined with other medications in 15 countries. Novel findings include an increase in combination therapy use over the decade of enrolment and the variability in treatment modalities between regions that was observed. Future studies should prospectively evaluate combination therapy in a randomized fashion in patients with acromegaly, ideally using central standardized MRI reading and laboratory measurements.

## Supplementary Material

Supporting Table 1

Supporting Table 2

Supporting Table 3

Supporting Table 4

## Declaration of interest

C J S received honoraria from Pfizer as a member of the ACROSTUDY Steering Committee as well as speakers and consultancy fees from Pfizer, Ipsen, Novartis, Chiasma and Strongbridge. B M K B received honoraria from Pfizer as a member of the ACROSTUDY Steering Committee, has served as PI of research grants from Novartis to Massachusetts General Hospital and has received occasional consulting honoraria from Chiasma, Ipsen and Novartis. P W is a former employee of Pfizer Inc. A M, F A and J H-H are fulltime employees of Pfizer Inc. ACROSTUDY is sponsored by Pfizer Inc.

## Funding

This research did not receive any specific grant from any funding agency in the public, commercial or not-for-profit sector.
