# Supplementary material for: Increasing frequency of combination medical therapy in the treatment of acromegaly with the GH receptor antagonist pegvisomant
Source: Eur J Endocrinol. 2018 Jan 25;178(4):321–9. doi: 10.1530/EJE-17-0996 (PMC5863474; doi:10.1530/EJE-17-0996)
Supplement: Supporting Table 1 [file eje-178-321-t001.pdf]

Supplementary Table 1. Total number of patients (All) and number and percentage of patients with history of no switch of treatment (‘No switch’) by visit since pegvisomant start on Combo SSA, Combo DA and Peg mono, respectively.

Years since peg start	Combo SSA			Combo DA			Peg mono			Total[§§§]		
	**Number of patients by medical treatment modality**											
	All[§]	No switch[§§]	%	All[§]	No switch[§§]	%	All[§]	No switch[§§]	%	All[§]	No switch[§§]	%
0	768	768	100	123	123	100	1128	1128	100	2019	2019	100
1	607	559	92	98	76	78	999	882	88	1704	1517	89
2	547	443	81	104	60	58	901	740	82	1552	1243	80
3	475	341	72	109	55	50	796	628	79	1380	1024	74
4	381	241	63	93	43	46	673	505	75	1147	789	69
5	313	175	56	69	31	45	570	395	69	952	601	63
6	251	123	49	56	22	39	458	307	67	765	452	59
7	195	85	44	39	15	38	337	216	64	571	316	55
8	136	58	43	28	8	29	215	148	69	379	214	56
9	80	30	38	12	2	17	127	90	71	219	122	56
10	38	11	29	3	0	0	60	45	75	101	56	55

[§]Total number of patients on respective treatment. [§§]Total number of patients with no history of switch of treatment at a specific year since peg start. %: percentage of patients at a specific year without a history of treatment switch. [§§§] Patients without Peg dose or on other treatment than presented are excluded from the table. (At pegstart it was 24 patients or 1.17%). Note also that different patients have different lengths of follow-up (From ‘peg start mono’) up to 10 years of follow-up). The column ‘All’ gives the number of patients with the specific number of years of follow-up (however, with potential history of switch). The ‘No switch’ column gives the corresponding number of patients who have consecutively stayed on the same treatment modality since peg start.
