# Supplementary material for: Increasing frequency of combination medical therapy in the treatment of acromegaly with the GH receptor antagonist pegvisomant
Source: Eur J Endocrinol. 2018 Jan 25;178(4):321–9. doi: 10.1530/EJE-17-0996 (PMC5863474; doi:10.1530/EJE-17-0996)
Supplement: Supporting Table 2 [file eje-178-321-t002.pdf]

**Supplementary Table 2.** Mean and SD of daily pegvisomant dose and average IGF-I/ULN by treatment and year since pegvisomant start. All available IGF-I data including data after possible switch of treatment modality.

| Years since peg start | Combo SSA | | | Combo DA | | | Peg mono | | | Total | | |
|---|---|---|---|---|---|---|---|---|---|---|---|---|
| | IGF-I<ULN | IGF-I>ULN | All* | IGF-I<ULN | IGF-I->ULN | All* | IGF-I<ULN | IGF-I>ULN | All* | IGF-I<ULN | IGF-I >ULN | All* |
| **Daily pegvisomant dose mg/day, Mean (SD)** | | | | | | | | | | | | |
| 0 | 8.9 (4.1) | 10.6 (8.4) | 10.1 (7.2) | 10.2 (2.3) | 12.1 (9.1) | 11.3 (7.3) | 11.6 (5.1) | 12.2 (8.2) | 12.3 (8.2) | 10.7 (4.8) | 11.6 (8.3) | 11.4 (7.8) |
| 1 | 13.4 (7.1) | 14.5 (8.9) | 13.6 (7.6) | 15.1 (6.9) | 15.2 (6.9) | 15.1 (6.3) | 15.6 (7.1) | 16.9 (7.4) | 15.8 (7.4) | 14.9 (7.2) | 15.9 (7.9) | 15.0 (7.5) |
| 2 | 14.0 (7.8) | 16.7 (9.3) | 14.5 (8.1) | 16.2 (5.3) | 20.2 (8.1) | 17.5 (7.2) | 16.3 (7.5) | 18.4 (8.2) | 16.8 (7.7) | 15.6 (7.5) | 17.9 (8.7) | 16.0 (7.9) |
| 5 | 17.9 (11.7) | 22.3 (12.0) | 17.9 (11.1) | 18.8 (7.3) | 19.9 (9.1) | 19.0 (9.1) | 17.6 (8.3) | 20.3 (10.0) | 18.0 (8.6) | 17.6 (9.1) | 20.9 (10.6) | 18.1 (9.6) |
| 7 | 21.3 (17.0) | 19.7 (10.9) | 19.4 (12.8) | 22.0 (6.2) | 28.6 (11.7) | 21.6 (9.3) | 15.9 (6.9) | 19.4 (9.5) | 18.1 (9.2) | 17.6 (10.2) | 20.3 (10.5) | 18.8 (10.6) |
| **IGF-I/ULN Mean (SD)** | | | | | | | | | | | | |
| 0 | | | 2.03 (1.15) | | | 1.95 (1.11) | | | 1.87 (0.96) | | | 1.93 (1.04) |
| 1 | | | 1.12 (0.71) | | | 1.13 (0.78) | | | 1.02 (0.62) | | | 1.06 (0.66) |
| 2 | | | 1.06 (0.56) | | | 1.06 (0.58) | | | 0.97 (0.51) | | | 1.00 (0.54) |
| 5 | | | 1.09 (0.64) | | | 1.14 (0.99) | | | 0.91 (0.53) | | | 0.98 (0.52) |
| 7 | | | 1.15 (0.58) | | | 0.99 (0.37) | | | 0.90 (0.49) | | | 0.98 (0.52) |

*Also including patients without IGF-I measurements.
