# Supplementary material for: Increasing frequency of combination medical therapy in the treatment of acromegaly with the GH receptor antagonist pegvisomant
Source: Eur J Endocrinol. 2018 Jan 25;178(4):321–9. doi: 10.1530/EJE-17-0996 (PMC5863474; doi:10.1530/EJE-17-0996)
Supplement: Supporting Table 3 [file eje-178-321-t003.pdf]

Supplementary Table 3. Number of patients with IGF-I measurement and number and percentage of patients with normal IGF-I value (IGF-I<ULN) by year since peg start and treatment category. IGF-I data at and after any treatment category switch are excluded.

	Number of patients by medical treatment category											
**Years since pegstart**	**Combo SSA**			**Combo DA**			**Peg mono**			**Total[§§§]**		
	All[§]	Normal[§§]	%	All[§]	Normal[§§]	%	All[§]	Normal[§§]	%	All[§]	Normal[§§]	%
0	471	51	10.8	88	13	14.8	817	107	13.1	1376	171	12.4
1	337	183	54.3	55	29	52.7	596	366	61.4	988	578	58.5
2	266	155	58.3	48	30	62.5	523	347	66.4	837	532	63.6
3	185	117	63.2	43	24	55.8	421	279	66.3	649	420	64.7
4	120	74	61.7	27	17	63.0	345	225	65.2	492	316	64.2
5	66	40	60.6	21	16	76.2	263	191	72.6	350	247	70.6
6	44	27	61.4	9	5	55.6	183	123	67.2	236	155	65.7
7	31	13	41.9	7	5	71.4	120	85	70.8	158	103	65.2

[§]Total number of patients on respective treatment with IGF-I measurement reported. [§§]Total number of patients with normal IGF-I. %: percentage of patients with normal IGF-I (IGF-I < ULN). [§§§] Patients without an IGF-I measurement, Pegvisomant dose or on other treatment than presented, and data at or after switch of treatment are excluded from the table.
