# Supplementary material for: Increasing frequency of combination medical therapy in the treatment of acromegaly with the GH receptor antagonist pegvisomant
Source: Eur J Endocrinol. 2018 Jan 25;178(4):321–9. doi: 10.1530/EJE-17-0996 (PMC5863474; doi:10.1530/EJE-17-0996)
Supplement: Supporting Table 4 [file eje-178-321-t004.pdf]

Supplementary Table 4. Summary of pituitary tumor imaging results from last MRI during combo SSA combo DA or Peg mono exposure periods combined.

Local versus Central reading. Data for last MRI was retrieved at least 30 days after the first reading (before pegvisomant start).

Local MRI reading			Central MRI reading result, No.					
Result	Total No	(%)	Increased	Decreased	Increased and decreased	No change	Insufficient data	Not done
Increase only, at least once	119	(7.2)	20	7	3	20	6	63
Decreased only, at least once	323	(19.6)	7	48	1	24	15	228
Both increased and decreased	54	(3.3)	13	10	3	5	5	18
No change	1145	(69.5)	2	9	1	19	4	1110
Missing	6	(0.4)	0	1	2	3	0	0
Total	1647	(100.0)	42	75	10	71	30	1419
